# Dependence on nicotine in US high school students in the context of changing patterns of tobacco product use

**DOI:** 10.1111/add.15403

**Published:** 2021-01-22

**Authors:** Sarah E. Jackson, Jamie Brown, Martin J. Jarvis

**Affiliations:** ^1^ Department of Behavioural Science and Health University College London London UK

**Keywords:** Adolescents, dependence, e‐cigarettes, nicotine, smoking, tobacco products

## Abstract

**Background and Aim:**

There have been substantial recent changes in youth tobacco product use in the United States—including, notably, a rapid increase in use of e‐cigarettes. It is not known whether, and if so how far, these changes are reflected in levels of nicotine dependence. This study used data from a large, nationally representative sample of US adolescents to (i) estimate the annual prevalence of nicotine dependence in relation to current use of tobacco products, (ii) describe trends in dependence over time and (iii) evaluate whether the increase in youth use of tobacco products has been paralleled by a similar increase in the population burden of nicotine dependence.

**Design:**

Secondary analysis of National Youth Tobacco Surveys conducted annually, 2012–19.

**Setting:**

United States.

**Participants:**

A total of 86 902 high school students.

**Measurements:**

Prevalence of (i) strong cravings to use tobacco in the past 30 days and (ii) wanting to use nicotine products within 30 minutes of waking, in relation to type of product used (cigarettes, other combustible tobacco, smokeless tobacco, e‐cigarettes).

**Findings:**

Between 2012 and 2019 there was a marked decline in past 30‐day cigarette smoking and a surge in use of e‐cigarettes. Different products were associated with differing levels of nicotine dependence, with cigarettes characterized by highest dependence (strong craving 42.3%; wanting to use within 30 minutes 16.8% among exclusive users in 2019) and e‐cigarettes in otherwise tobacco‐naive students by low dependence (16.1 and 8.8% respectively in 2019). The overall 33.8% increase in population use of nicotine products between 2012 and 2019 (from 23.2 to 31.2%) was not accompanied by an equivalent increase in overall population burden of dependence {percentage reporting craving 10.9% [95% confidence interval (CI) = 9.8–12.2%] in 2012 and 9.5% (95% CI = 7.5–12.0%) in 2019; wanting to use within 30 minutes 4.7% (95% CI = 4.0–5.5%) in 2012, 5.4% (95% CI = 4.0–7.2%) in 2019}.

**Conclusions:**

Among US high school students, increases in the prevalence of nicotine product use from 2012 to 2019 do not appear to have been accompanied by a similar increase in the population burden of nicotine dependence. This may be at least partly attributable to a shift in the most common product of choice from cigarettes (on which users are most dependent) to e‐cigarettes (on which users are least dependent).

## Introduction

Globally, cigarette smoking is a leading public health concern. Smoking is typically initiated and established in adolescence [1], tracks strongly across the life‐course [2] and is associated with increased risk of disease, disability and premature death [3]. Cigarettes are highly addictive, leading in many cases to great difficulty in achieving and maintaining abstinence. For these reasons, preventing the uptake of cigarette smoking is a priority. In the United States, concerns about youth smoking extends beyond cigarettes to a range of other tobacco and nicotine products. The Centers for Disease Control and Prevention (CDC) emphasize the critical role of preventing tobacco product use among youth in ending the US tobacco epidemic [4].

While data from the National Youth Tobacco Survey (NYTS) have shown a decline in youth tobacco use in recent years, reports of an increase in prevalence of use of any tobacco product among high school students in 2018 (up 38.3% on 2017 estimates, from 19.6 to 27.1%) provided cause for concern [5, 6]. However, there was substantial variation in patterns of prevalence among different types of tobacco product. A wide variety of tobacco products are available in the United States, including cigarettes, other combustible tobacco (e.g. cigars, cigarillos, bidis, hookahs), smokeless tobacco (e.g. chewing tobacco, snuff, dip) and e‐cigarettes. Although e‐cigarettes are not widely classified as—and do not contain—tobacco [7], many contain nicotine and they are categorized as a tobacco product in the United States under a court‐endorsed legal framework for Food and Drug Administration (FDA) regulation. It is e‐cigarettes that are the driving force behind the rising prevalence of youth use of tobacco products, with past 30‐day use of e‐cigarettes by high school students increasing from 11.7% in 2017 to 20.8% and 27.5% in 2018 and 2019, respectively [5, 8]. Meanwhile, use of cigarettes (the next most popular product category) declined to a historical low of 5.8% in 2019 [8].

In terms of their potential impact on health, a key feature of tobacco products, including e‐cigarettes, is their tendency to result in dependence. Nicotine, present in tobacco products, binds to nicotinic acetylcholine receptors in the ventral tegmental area of the brain and triggers the release of dopamine in the nucleus accumbens. Dopamine acts as a neural ‘teaching signal’, which causes the brain to form an association between the current situation and the impulse to engage in whatever action immediately preceded this release (e.g. smoking). Repeated absorption of nicotine from cigarettes is known to cause changes to the functioning of the ventral tegmental area and nucleus accumbens: when levels of nicotine in the brain are lower than usual there is an abnormally low level of neural activity in these regions [9]. This leads to feelings of need (‘craving’) for nicotine to restore normal functioning. As a result of neural adaptation to the presence of nicotine in the brain, many smokers experience nicotine withdrawal symptoms (e.g. irritability, restlessness, difficulty concentrating) within a few hours of abstinence from cigarettes [9, 10, 11]. Thus, dependence is characterized by cravings, feeling a need to use tobacco and withdrawal symptoms during periods of abstinence. Understanding how far different tobacco product categories—which provide different nicotine concentrations and methods of nicotine delivery—differ in the levels of dependence experienced by users, and how changing patterns of use of these products have affected the overall prevalence or burden of nicotine dependence among youth, is important in evaluating the ‘big picture’ of youth tobacco use in the United States and its implications for public health.

Surveys of college students indicate that young people who use tobacco products perceive e‐cigarettes to be less addictive than cigarettes [12]. This perception may reflect how the nicotine delivery profile of e‐cigarette devices varies considerably, with some devices providing much less than cigarettes [13]. Also, inexperienced users are typically able to obtain less nicotine from e‐cigarettes [13]. It has also been suggested that it may be attributable to e‐cigarette marketing claims or users not ‘feeling’ addicted or experiencing cravings [12]. This is corroborated by comparisons of cravings and withdrawal symptoms among adult users of different tobacco product categories, which show weaker evidence of dependence among exclusive e‐cigarette users than exclusive cigarette smokers, and the highest levels of dependence among dual users of cigarettes and e‐cigarettes [14, 15]. Whether the patterns of dependence across tobacco product categories are similar in youth is not known.

In this paper we use data from the NYTS between 2012 and 2019 to analyse indicators of nicotine dependence in relation to current use of tobacco products and e‐cigarettes among US high school students. We also report on time trends in the overall burden of nicotine dependence in this population and compare these with time trends in youth use of tobacco products. Specifically, we aimed to address the following research questions:

1. Between 2012 and 2019, what was the population burden of nicotine dependence, indexed by prevalence of (i) strong cravings to use tobacco in the past 30 days and (ii) wanting to use nicotine products within 30 minutes of waking, among US high school students who, in the past 30 days, had used (a) no tobacco product, (b) e‐cigarettes only, (c) smokeless (but no combustible) tobacco, with/without e‐cigarettes, (d) combustible tobacco (but no cigarettes), with/without e‐cigarettes and (e) cigarettes, with/without e‐cigarettes?
2. What function best describes trends in the population burden of nicotine dependence among US high school students between 2012 and 2019?
3. Between 2012 and 2019, was the substantial increase in the prevalence of tobacco product use among US high school students paralleled by a comparable increase in the population burden of nicotine dependence?

## Method

### Design

Cross‐sectional analysis of data from the National Youth Tobacco Survey (NYTS) collected annually between 2012 and 2019.

### Data

The NYTS is designed to produce a nationally representative cross‐sectional sample of students from US middle and high schools. It was developed to inform national and state tobacco prevention and control programmes. Full details of the NYTS methodology are available elsewhere [16]. Briefly, a three‐stage cluster sampling procedure is used to generate a nationally representative sample of students in grades 6–12. An anonymous, self‐administered questionnaire is used to collect data on a range of variables relevant to tobacco use. Until 2018 the survey was administered in paper‐and‐pencil form, with a switch to digital in 2019. For the present analysis, we used data on high school students (grades 9–12) collected between 2012 and 2019, as these surveys have included questions on nicotine dependence. Throughout this period, a total of 86 902 high school students completed the survey, with overall annual response rates (calculated as the product of the school‐ and student‐level participation rates) ranging between 63.4 and 73.6%.

### Measures

#### Measurement of tobacco product use

Current use of tobacco products was defined as any use of the following four product categories in the past 30 days: cigarettes, other combustible tobacco (defined as cigars, cigarillos, little cigars, pipes, bidis, hookahs), non‐combustible tobacco (chewing tobacco, snuff, dip, snus and dissolvable tobacco) and e‐cigarettes.

#### Measurement of nicotine dependence

Nicotine dependence was assessed using measures of craving and time to wanting to first use tobacco products after waking. These questions were asked in the context of survey guidance that made it explicitly clear to respondents that tobacco products included e‐cigarettes: ‘The next six sections of questions ask about your use of particular kinds of tobacco products, such as cigarettes, cigars, smokeless tobacco, electronic cigarettes, hookahs, pipes, snus, dissolvable tobacco, and bidis’.

Craving was assessed with the question: ‘During the past 30 days, have you had a strong craving or felt like you really needed to use a tobacco product of any kind?’, with ‘yes/no’ response options. This item was first included in the 2012 NYTS and has been asked in every year since 2014. In 2012 and 2014 examples were given at the end of the question: in 2012, ‘such as smoking a cigarette or using chewing tobacco, snuff, dip, or snus’ and in 2014, ‘such as smoking a cigarette or cigar, or using chewing tobacco’. No examples were given in subsequent surveys.

Time to wanting to first use tobacco products after waking was assessed with the question: ‘How soon after you wake up do you want to use a tobacco product?’. This item was first included in the 2012 NYTS and has been asked in every year subsequently. Response options in 2012 and 2013 were: (i) ‘I do not use tobacco’, (ii) ‘within 5 minutes’, (iii) ‘from 6 to 30 minutes’, (iv) ‘from more than 30 minutes to 1 hour’, (v) ‘after more than 1 hour but less than 24 hours’ and (vi) ‘I rarely want to use tobacco’. In 2014, the first response option was changed from ‘I do not use tobacco’ to ‘I do not want to use tobacco’, and the revised wording has been used in all subsequent surveys. For our analysis, we dichotomized responses to reflect wanting to first use tobacco products within 30 minutes of waking [responses (ii) and (iii) versus all other options], a commonly used and validated indicator of nicotine dependence among adults [17]. Because youth users may have greater constraints on their ability to use within this time‐frame, we ran sensitivity analyses using a dichotomy that reflected wanting to first use within 60 minutes of waking [responses (ii), (iii) and (iv) versus all other options].

### Statistical analysis

#### Prevalence estimates

We used the complex survey analysis module in SPSS version 24 to adjust for the sampling design of the survey and to generate estimates [with 95% confidence intervals (CI)] of nicotine dependence (indexed by past 30‐day craving and wanting to first use tobacco products within 30 minutes of waking) in relation to current use of tobacco products and survey year. As a sensitivity analysis, we also estimated the prevalence of wanting to first use tobacco products within 60 minutes of waking.

We defined the population burden of nicotine dependence as the overall population average dependence score (for either craving or wanting to first use within 30 minutes of waking) among all participants, including those who reported not having used any product in the past 30 days.

#### Trend analysis

Using R version 3.6.3, we conducted a trend analysis of population‐level indices of dependence during the study period (2012–19). First, time (survey year) was regressed onto (i) prevalence of past 30‐day craving and (ii) wanting to first use tobacco products within 30 minutes of waking in a simple linear regression model. Next, several additional models were assessed: (1) quadratic, (2) cubic, (3) logarithmic (level‐log model), (4) exponential (log‐level model) and (5) power (log–log model). Other functions (e.g. quartic and quantic polynomial regressions) were not tested, as we did not believe they would reflect plausible underlying trends in prevalence and could lead to overfitting. Because craving was not assessed in 2013, the missing value was imputed with the mean of 2012 and 2014.

To identify the best overall models, all the resulting regression models were compared using the Akaike information criterion (AIC) as the primary measure of fit and the adjusted *R* ^2^ and Bayesian information criterion (BIC) as secondary measures of fit. In general, the smaller the AIC and BIC and the larger the adjusted *R* ^2^, the better the model fit. A prerequisite in using the AIC and BIC to compare models is that the dependent variable is on the same scale; thus, to ensure equivalence for the exponential trend and power trend models, a correction was applied to the AIC and BIC. This involved adding the Jacobian of the log transformation; that is $2\sum_{i}\log\left(y_{i}\right)$, where *y* is the outcome variable of interest. Model fit indices for each model are shown in Supporting information, Table S1. We report results of the linear and best fitting models in Supporting information, Table S2.

#### Comparison of changes in dependence against the increase in tobacco product use

To assess whether changes in youth use of tobacco products over the study period were paralleled by similar changes in the population burden of nicotine dependence, we aimed to compare estimates from the best‐fitting models for dependence with corresponding results for product use. Because the best‐fitting model for each variable (both measures of dependence and product use) was cubic (Supporting information, Tables S1 and S3), and it is difficult to meaningfully compare cubic parameters (which include separate estimates for linear, quadratic and cubic components), we used piece‐wise regression with a break‐point at the point of inflexion (2017) to approximate trends in a way that can more easily be compared and interpreted. This model provided a good fit for the data (Supporting information, Table S4); results are shown in Supporting information, Table S5. In separate regression models, we then compared slopes before and after the 2017 break‐point for (i) past 30‐day craving versus past 30‐day product use and (ii) wanting to first use tobacco products within 30 minutes of waking versus past 30‐day product use. For each slope, we tested the interaction between variable (dependence versus product use) and year on prevalence.

The analyses were not pre‐registered and so the results should be considered exploratory.

## Results

**Figure 1:**
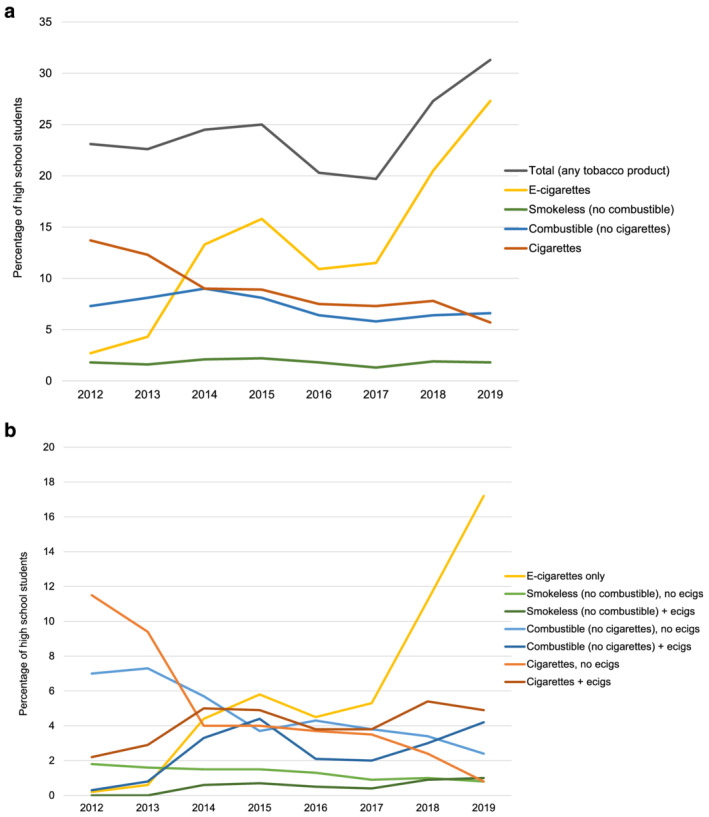
Past 30‐day tobacco product use among high school students, 2012–19: (a) across the four main product categories and (b) stratified by use of e‐cigarettes. [Colour figure can be viewed at wileyonlinelibrary.com]

Between 2012 and 2019, patterns of tobacco product use changed substantially among high school students. The prevalence of past 30‐day use of any tobacco product increased from 23.2 to 31.2%. The prevalence of past 30‐day use of e‐cigarettes increased dramatically, use of cigarettes declined and use of combustible (non‐cigarette) and smokeless tobacco was relatively stable (Fig. 1a). Among users of cigarettes and other combustible tobacco, the proportion who used e‐cigarettes increased considerably, with dual use of cigarettes and e‐cigarettes becoming more common than exclusive cigarette use from 2014, although there was little change in the prevalence of e‐cigarette use among users of smokeless tobacco (Fig. 1b).

**Figure 2:**
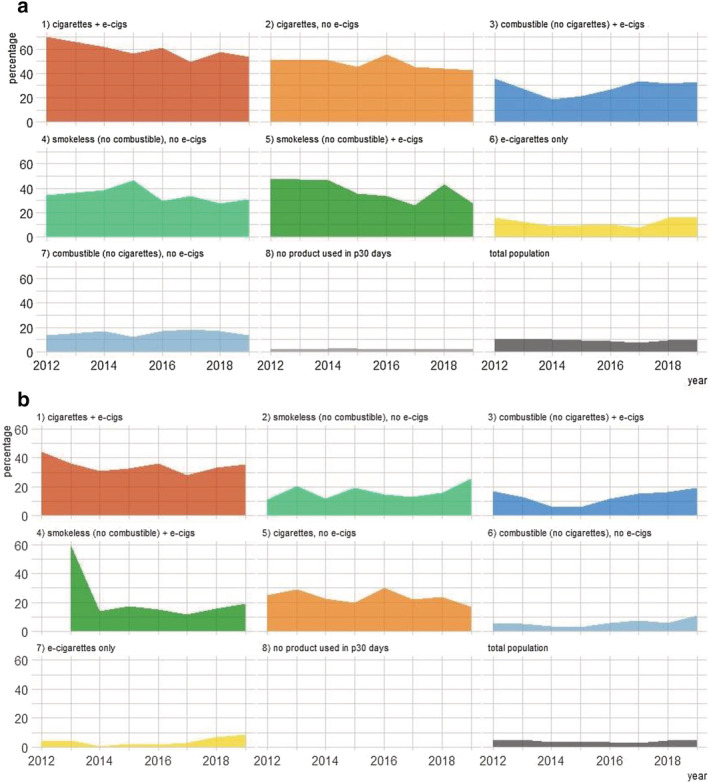
Percentage of different groups of high school students reporting (a) strong craving for tobacco in past 30 days and (b) wanting to first use tobacco products within 30 minutes of waking, by type of product used in the past 30 days. Panels are ranked in order of prevalence in 2019. [Colour figure can be viewed at wileyonlinelibrary.com]

Measures of nicotine dependence varied substantially by type of product used in the past 30 days (Fig. 2). With the exception of 2013, at each time‐point the highest point estimates for dependence were seen in dual users of cigarettes and e‐cigarettes. In 2019, 53.8% of dual cigarette and e‐cigarette users reported strong craving for tobacco in the past 30 days and 35.5% reported wanting to first use tobacco within 30 minutes of waking. High levels of dependence were also observed among cigarette smokers who did not use e‐cigarettes (42.3 and 16.8%, respectively, in 2019). Among those who used any tobacco product in the past 30 days, the lowest levels of dependence were observed among those who used e‐cigarettes only (16.1 and 8.8% in 2019) or combustible (non‐cigarette) tobacco without e‐cigarettes (13.3 and 11.1% in 2019). Wanting to first use tobacco within 60 minutes of waking followed the same pattern (Supporting information, Table S6).

**Figure 3:**
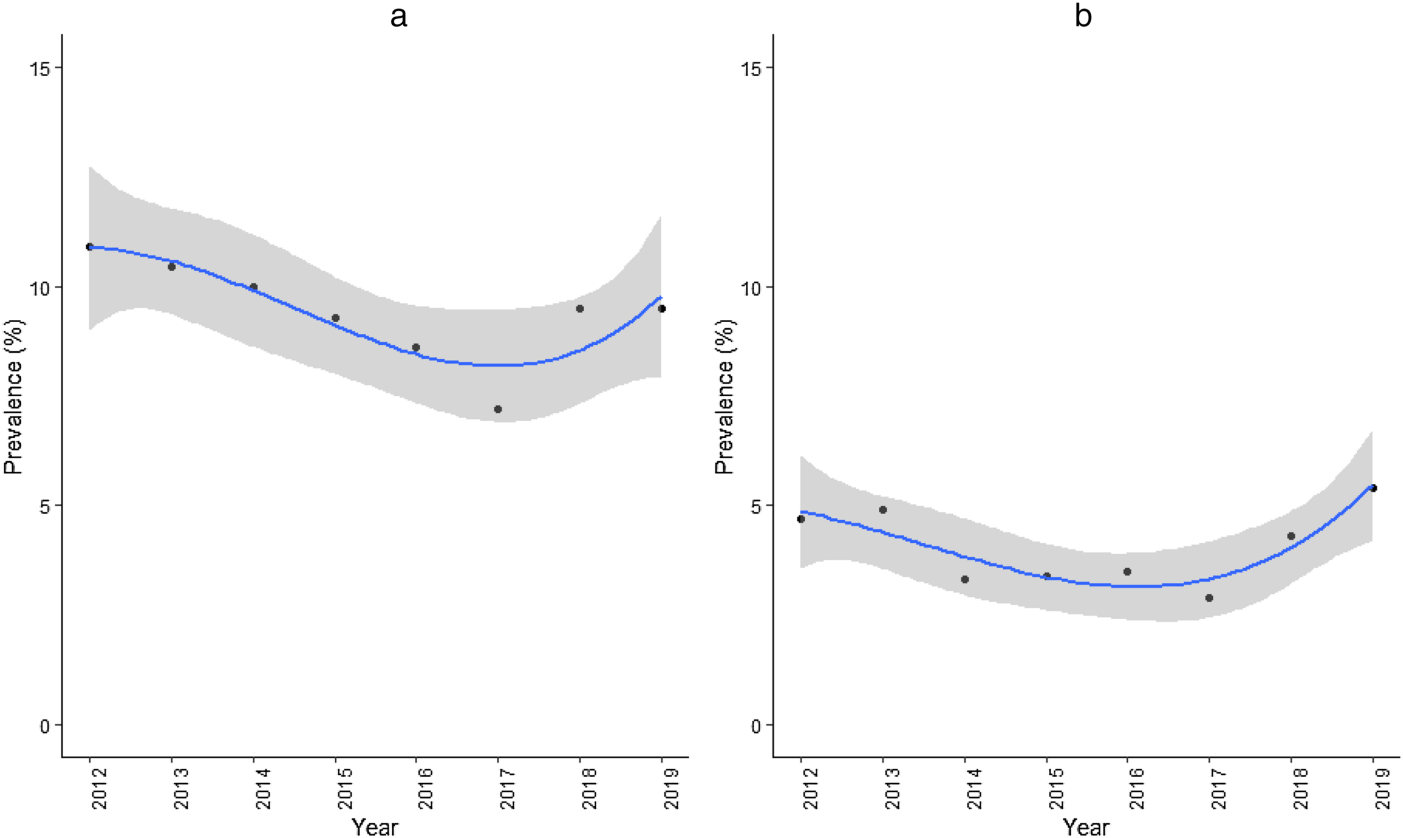
Best‐fitting (cubic) models for the percentage of high school students reporting (a) strong craving for tobacco in past 30 days and (b) wanting to first use tobacco products within 30 minutes of waking, 2012–19. Lines represent modelled trends; shaded bands represent 95% confidence intervals. [Colour figure can be viewed at wileyonlinelibrary.com]

Despite the considerable changes in product use and the association between product use and nicotine dependence, changes in the population burden of nicotine dependence during the study period were modest (Fig. 3). The best‐fitting regression models for both craving and wanting to first use tobacco within 30 minutes of waking were cubic trend models (Supporting information, Tables S1 and S2). The proportion of all high school students reporting strong craving for tobacco in the past 30 days fell from 10.9% in 2012 to 7.2% in 2017, then increased to 9.5% in 2018 and 2019 (Table 1, Fig. 3a). A similar pattern was seen for reports of wanting to first use tobacco products within 30 minutes of waking (4.7% in 2012, 2.9% in 2017, 5.4% in 2019; Table 2, Fig. 3b). Between 2012 and 2017, the slopes for measures of dependence (using piece‐wise regression) did not differ significantly from the slope for tobacco product use, indicating a similar change in prevalence over time (Supporting information, Table S7). However, between 2017 and 2019, the slope for wanting to first use tobacco within 30 minutes of waking was significantly less steep than the slope for tobacco product use (β = –4.55, 95% CI = –9.04 to −0.06), indicating a smaller increase in prevalence over time. A similar difference was evident between past 30‐day craving and past 30‐day tobacco product use, although it did not reach statistical significance (β *=* –4.65, 95% CI = –9.96 to 0.66).

**Table 1:** Percentage (with 95% confidence interval) of high school students reporting strong craving for tobacco in past 30 days by type of product used in past 30 days. Figures in italics give the estimated percentage of all high school students using that product in past 30 days

	No product used in p30 days	e‐cigarettes only	Smokeless, but no combustible		Combustible, no cigarettes		Cigarettes		Total
			No e‐cigs	+ e‐cigs	No e‐cigs	+ e‐cigs	No e‐cigs	+ e‐cigs	
2012 (*n* = 12 899)	2.3 (2.0–2.7)	16.0 (5.5–38.3)	34.1 (26.8–42.2)	47.7 (26.0–70.4)	13.4 (10.6–16.8)	36.1 (22.3–52.7)	51.0 (48.0–54.1)	70.0 (64.7–74.7)	10.9 (9.8–12.2)
% population	*76.8 (75–79)*	*0.3 (0.2–0.4)*	*1.8 (1.4–2.4)*	*0.0 (0.0–0.1)*	*7.1 (6.3–7.9)*	*0.3 (0.2–0.5)*	*11.5 (10.2–12.8)*	*2.2 (1.7–2.7)*	
2014 (*n* = 10 190)	2.6 (2.2–3.1)	8.9 (6.0–13.1)	38.4 (31.4–45.9)	46.6 (31.2–62.6)	16.9 (13.3–21.3)	18.9 (14.0–25.0)	51.1 (46.3–55.9)	61.5 (55.7–66.9)	10.0 (8.9–11.1)
% population	*75.5 (73–78)*	*4.4 (3.3–5.7)*	*1.5 (1.2–2.0)*	*0.6 (0.4–0.8)*	*5.8 (4.9–6.7)*	*3.3 (2.7–4.0)*	*4.0 (3.2–4.9)*	*5.0 (4.2–6.0)*	
2015 (*n* = 9433)	2.5 (2.0–3.0)	9.4 (6.3–13.8)	46.4 (36.0–57.1)	35.9 (22.7–51.7)	12.1 (9.0–15.9)	21.2 (16.0–27.4)	45.4 (38.6–52.5)	56.0 (50.6–61.2)	9.3 (8.0–10.7)
% population	*75.0 (73–77)*	*5.8 (4.8–7.0)*	*1.6 (1.1–2.3)*	*0.7 (0.5–0.9)*	*3.8 (3.2–4.4)*	*4.4 (3.7–5.1)*	*4.0 (3.2–5.1)*	*4.8 (4.0–5.9)*	
2016 (*n* = 10 897)	2.3 (1.9–2.8)	10.3 (7.5–13.9)	29.5 (20.7–40.1)	33.4 (19.4–51.1)	17.3 (14.3–20.8)	26.8 (20.5–34.3)	55.7 (49.2–62.9)	60.8 (53.0–68.2)	8.6 (7.5–9.9)
% population	*79.7 (78–82)*	*4.5 (3.7–5.4)*	*1.3 (1.0–1.7)*	*0.5 (0.4–0.8)*	*4.3 (3.7–5.0)*	*2.1 (1.8–2.6)*	*3.7 (2.9–4.7)*	*3.9 (3.2–4.7)*	
2017 (*n* = 10 186)	1.9 (1.5–2.3)	7.7 (5.1–11.5)	33.7 (20.1–50.7)	25.8 (11.9–47.4)	18.1 (13.8–23.3)	33.8 (22.7–47.0)	45.7 (38.9–52.7)	49.4 (42.9–56.0)	7.2 (6.2–8.3)
% population	*80.2 (78–83)*	*5.3 (4.2–6.6)*	*0.9 (0.7–1.3)*	*0.4 (0.2–0.6)*	*3.9 (3.3–4.5)*	*2.0 (1.5–2.8)*	*3.5 (2.9–4.3)*	*3.8 (3.2–4.6)*	
2018 (*n* = 10 991)	1.9 (1.5–2.4)	15.8 (12.9–19.1)	27.1 (19,4–36.6)	43.1 (30.4–56.8)	17.3 (12.6–23.4)	31.5 (26.5–36.9)	43.8 (36.1–51.8)	58.0 (53.4–62.5)	9.5 (8.5–10.7)
% population	*72.7 (71–75)*	*11.2 (9.6–13.1)*	*1.0 (0.7–1.3)*	*0.9 (0.7–1.3)*	*3.4 (2.8–4.1)*	*3.0 (2.5–3.5)*	*2.4 (2.0–2.9)*	*5.4 (4.6–6.4)*	
2019 (*n* = 10 097)	2.1 (0.9–5.2)	16.1 (13.5–19.1)	30.9 (17.1–49.2)	27.4 (17.4–40.0)	13.3 (9.3–18.7)	33.2 (27.9–38.8)	42.3 (31.1–54.3)	53.8 (47.0–60.5)	9.5 (7.5–12.0)
% population	*68.7 (66–71)*	*17.2 (15.6–19.0)*	*0.8 (0.5–1.2)*	*1.0 (0.7–1.4)*	*2.4 (1.8–3.2)*	*4.2 (3.7–4.8)*	*0.8 (0.6–1.2)*	*4.9 (3.8–6.3)*	

**Table 2:** Percentage (with 95% confidence interval) of high school students reporting wanting to first use tobacco products within 30 minutes of waking by type of product used in past 30 days. Figures in italics give the estimated percentage of all high school students using that product in past 30 days

	No product used in p30 days	e‐cigarettes only	Smokeless, but no combustible		Combustible, no cigarettes		Cigarettes		Total
			No e‐cigs	+ e‐cigs	No e‐cigs	+ e‐cigs	No e‐cigs	+ e‐cigs	
2012 (*n* = 12 899)	0.2 (0.1–0.3)	4.0 (0.5–25.4)	11.3 (6.9–17.8)		6.0 (4.1–8.7)	17.0 (5.9–40.0)	25.2 (22.0–28.8)	44.4 (37.2–51.9)	4.7 (4.0–5.5)
% population	*76.9 (75–79)*	*0.2 (0.1–0.4)*	*1.8 (1.4–2.4)*	*0.0 (0.0–0.1)*	*7.0 (6.3–7.9)*	*0.3 (0.2–0.5)*	*11.5 (10.2–12.8)*	*2.2 (1.7–2.7)*	
2013 (*n* = 10 190)	0.4 (0.3–0.5)	4.6 (1.4‐4.3)	20.6 (14.0–29.2)	59.4 (21.4–88.7)	5.4 (3.6–8.1)	12.7 (4.0–33.6)	28.9 (24.7–33.6)	36.1 (29.8–43.0)	4.9 (4.1–5.9)
% population	*77.4 (76–79)*	*0.6 (0.5–0.8)*	*1.6 (1.1–2.2)*	*0.0 (0.0–0.1)*	*7.3 (6.5–8.2)*	*0.8 (0.6–1.1)*	*9.4 (8.2–10.7)*	*2.9 (2.4–3.5)*	
2014 (*n* = 11 399)	0.2 (0.1–0.4)	0.6 (0.2–1.4)	11.7 (7.3–18.2)	14.0 (7.9–23.7)	3.7 (2.5–5.5)	6.5 (4.0–10.5)	22.5 (17.5–28.5)	31.2 (26.8–36.0)	3.3 (2.8–4.0)
% population	*75.5 (73–78)*	*4.4 (3.3–5.7)*	*1.5 (1.2–2.0)*	*0.6 (0.4–0.8)*	*5.7 (4.9–6.7)*	*3.3 (2.7–4.0)*	*4.0 (3.2–4.9)*	*5.0 (4.2–6.0)*	
2015 (*n* = 9433)	0.2 (0.1–0.3)	2.1 (1.0–4.3)	19.2 (12.8–27.8)	17.4 (7.2–36.6)	2.7 (1.5–4.9)	5.8 (3.5–9.4)	20.0 (16.0–24.7)	32.6 (27.5 (38.1)	3.4 (2.7–4.2)
% population	*75.0 (73–77)*	*5.8 (4.8–7.0)*	*1.5 (1.1–2.3)*	*0.7 (0.5–0.9)*	*3.7 (3.2–4.4)*	*4.4 (3.7–5.1)*	*4.0 (3.2–5.1)*	*4.9 (4.0–5.9)*	
2016 (*n* = 10 991)	0.2 (0.1–0.2)	1.7 (0.7–3.9)	14.7 (7.3–27.3)	15.3 (5.5–36.0)	6.0 (4.0–9.0)	11.7 (7.6–17.5)	30.0 (23.3–37.7)	36.0 (30.1–42.4)	3.5 (2.7–4.4)
% population	*79.7 (78–82)*	*4.5 (3.7–5.4)*	*1.3 (1.0–1.7)*	*0.5 (0.5–0.8)*	*4.3 (3.7–5.0)*	*2.1 (1.8–2.6)*	*3.7 (2.9–4.7)*	*3.8 (3.2–4.6)*	
2017 (*n* = 10 186)	0.2 (0.1–0.3)	3.0 (1.4–6.2)	12.8 (6.8–22.9)	11.5 (3.1–34.9)	7.6 (4.1–13.8)	15.2 (9.0–24.3)	22.0 (16.4–28.9)	28.0 (22.7–34.0)	2.9 (2.3–3.6)
% population	*80.3 (78–83)*	*5.3 (4.2–6.6)*	*0.9 (0.7–1.3)*	*0.4 (0.2–0.6)*	*3.8 (3.3–4.5)*	*2.0 (1.5–2.8)*	*3.5 (2.9–4.3)*	*3.8 (3.2–4.6)*	
2018 (*n* = 10 991)	0.2 (0.1–0.4)	7.1 (4.9–10.2)	15.7 (9.1–25.8)	16.0 (9.0–27.0)	6.0 (3.4–10.3)	16.4 (12.5–21.1)	23.9 (17.7–31.3)	33.0 (28.6–37.8)	4.3 (3.7–5.0)
% population	*72.7 (71–75)*	*11.2 (9.6–13.1)*	*1.0 (0.7–1.3)*	*0.9 (0.7–1.3)*	*3.4 (2.8–4.1)*	*3.0 (2.5–3.5)*	*2.4 (2.0–2.9)*	*5.4 (4.6–6.4)*	
2019 (*n* = 10 097)	0.7 (0.1–4)	8.8 (7–11)	26.0 (13–46)	19.0 (12–29)	11.1 (7–18)	19.1 (15–25)	16.8 (9–28)	35.5 (31–41)	5.4 (4.0–7.2)
% population	*68.7 (67–71)*	*17.2 (15.6–19.0)*	*0.8 (0.5–1.2)*	*1.0 (0.7–1.4)*	*2.4 (1.8–3.2)*	*4.2 (3.7–4.8)*	*0.8 (0.6–1.2)*	*4.9 (3.8–6.3)*	

## Discussion

Between 2012 and 2019, the prevalence of past 30‐day use of tobacco products among US high school students rose from 23.4 to 31.3%, an increase of one‐third (33.8%). During the same period, both craving and time to wanting to use followed cubic trends, showing an initial decline, bottoming out in 2017, before a subsequent return to 2012 levels in 2019. The fact that there has not been an overall increase from 2012 to 2019 in the population burden of nicotine dependence while the prevalence of tobacco product use has risen by a third can be attributed to changes in the patterns of product use over time. Our results indicate that the decline in use of cigarettes has seen a population‐level shift away from not only the most popular product, but also the one on which users are most dependent. Meanwhile, e‐cigarettes have made rapid gains in popularity and currently have the lowest associated levels of dependence.

The highest levels of nicotine dependence were observed among those students who used both cigarettes and e‐cigarettes during the past month. This is consistent with evidence that heavier smokers are more likely to take up vaping [18, 19, 20], potentially to provide a means of satisfying nicotine cravings in situations where cigarette use is not permitted (for example, because of legislation or parental disapproval). It also mirrors findings from the US Adult National Tobacco Survey, which showed that dual users were more likely than exclusive smokers to report withdrawal and craving symptoms [14]. The much lower levels of dependence among exclusive e‐cigarette users than cigarette smokers are also in line with previous research. For example, an analysis of data from the Population Assessment of Tobacco and Health Study, a nationally representative survey of adult tobacco use in the United States, found that e‐cigarette users reported a longer time to first use of the day after waking than cigarette smokers and were less likely to consider themselves addicted, had strong cravings, found it difficult to refrain from using their product in places where it was prohibited or felt that they really needed to use their product [15].

It has been suggested, based on top‐line results from the NYTS, that e‐cigarettes are causing an epidemic of youth nicotine addiction [6]. When the preliminary data were released in 2019, acting FDA Commissioner Ned Sharpless commented: ‘The tremendous progress we've made in reducing youth tobacco use in the US is jeopardized by this onslaught of e‐cigarette use. Nobody wants to see children becoming addicted to nicotine, and we will continue to use the full scope of our regulatory authority thoughtfully and thoroughly to tackle this mounting public health crisis’ [21]. However, the data from the 2019 NYTS actually indicate that the extent of dependence on tobacco products is much the same today as it was in 2012, when the prevalence of e‐cigarette use was very low. While there appears to have been an increase in nicotine dependence during the last 2 years compared with record lows, it is far from reaching epidemic proportions (see Fig. 2, ‘total’ population). In addition, the fact that reports of strong craving and wanting to use tobacco within 30 minutes of waking were stable from 2018 to 2019 is not suggestive of a continuing rise in youth nicotine dependence. Our comparison of changes in dependence from 2017 to 2019 against the dramatic increase in product use during the same period indicated that dependence had not increased to a similar extent to product use. However, it will be vital to review the data from 2020 carefully, although complicated with data collection affected by the COVID‐19 pandemic.

Importantly, the FDA's claim that e‐cigarettes are undermining progress towards reducing use of tobacco products in this age group [21, 22] fails to account for differences in the risk profile of different product categories. A growing body of evidence demonstrates that e‐cigarettes are much less harmful than cigarettes, exposing users to considerably lower levels of toxins and carcinogens while potentially (but not always) providing similar levels of nicotine [23, 24, 25, 26]. Parallels can be drawn with Sweden, where the overall prevalence of tobacco use among men is comparable to other European Union (EU) countries [27], but the majority use snus (a moist powdered smokeless tobacco product) rather than smoking cigarettes. Because snus carries a lower risk to health than cigarettes, the level of mortality attributable to tobacco among Swedish men is lower than in any other EU Member State [28], resulting in a lower health burden despite equivalent prevalence of tobacco use. That is not to say that nicotine dependence *per se* is entirely without risk: a body of evidence outlines risks of nicotine use on the adolescent brain [29] and increased incidence of health problems (e.g. lung cancer, chronic obstructive pulmonary disease), even after accounting for smoking behaviour and other risk factors [30, 31]. Nonetheless, while it is understandable that data indicating a rise in youth use of tobacco products may provide cause for concern, it is important to take into account the relative risks of the different product categories when evaluating progress, and consider that a population‐level shift from cigarette to e‐cigarette use will probably result in substantially reduced morbidity and mortality (the objective at the heart of ambitions to drive down youth tobacco use). There is widespread concern that e‐cigarettes may serve as a ‘gateway’ to smoking in adolescents [32, 33, 34, 35, 36]. It is clear from a number of longitudinal studies that young people who use e‐cigarettes are more likely to use cigarettes at a later date than those who do not. The direction of causality, however, is not clear [37, 38]. If the relationship was causal, high prevalence of e‐cigarette use would mean that adolescents who would not have otherwise used tobacco will be exposed to nicotine, develop nicotine dependence and as a result will be more likely to go on to use cigarettes and other products. It could be argued that such an effect would simply shift the burden of dependence on e‐cigarettes sooner or later. However, the NYTS data do not provide strong evidence of an effect of using e‐cigarettes on subsequent uptake and use of cigarettes and other combustible products. A previous analysis of e‐cigarette use among high school students in relation to life‐time history of use of tobacco products showed that, for the great majority of adolescents with any substantial cigarette smoking history, cigarettes were the first tobacco product tried, prior to use of e‐cigarettes [39]. In addition, there has been no sign of the rapid declines in the prevalence of cigarette smoking and trying combustible products being reversed through the upsurge of e‐cigarette use [40], and population‐level trend modelling suggests that e‐cigarettes are more likely to divert adolescents away from cigarette use than serve as a gateway [41]. Mendelian randomization suggests a common genetic vulnerability to both smoking and e‐cigarette use, which may reflect a broad risk‐taking phenotype [42]. While our analysis was not designed to test this gateway effect, our finding that use of e‐cigarettes was associated with lower levels of dependence than use of any other tobacco product we examined is also worth noting in this context.

Strengths of this analysis include the large, representative sample and triangulation on nicotine dependence through the use of two established measures; craving and time to first use. However, there were also limitations. First, use of tobacco products was defined according to self‐reports of any use in the past 30 days. This crude measure fails to capture frequency of use and, in the case of the categories that combined e‐cigarettes and other tobacco products, which product is used most often, both of which may influence dependence. Secondly, while guidance to students completing the survey explicitly defined e‐cigarettes as tobacco products in the same sense as cigarettes, cigars, cigarillos, pipes, smokeless tobacco and bidis—and measures of dependence asked participants to reflect on their experience with all the categories of tobacco products they had previously been questioned about—it is possible that some interpreted the questions about nicotine dependence as not applying to e‐cigarettes, leading to underestimation of dependence. Qualitative work is required to explore this possibility. It has been suggested that product‐specific measures of dependence are required to precisely capture the dependence produced by both nicotine and its administration forms, but this would make comparability across product categories difficult [43]. Thirdly, the data do not account for the fact that the e‐cigarette product category has changed substantially over the study period, whereas cigarettes have not. As such, contrasts of levels of dependence among e‐cigarette users in 2019 versus 2012 are not comparing like with like. In addition, the data do not distinguish between nicotine and non‐nicotine e‐cigarettes, meaning that we were unable to ascertain the proportion of e‐cigarette users who used nicotine‐free products. Finally, the correlational design means that it is not possible to determine cause and effect. While we speculate that nicotine dependence is more common among dual users of cigarettes and e‐cigarettes because cigarette smokers who are more dependent may be more likely to take up e‐cigarette use to use nicotine in situations where smoking is not permitted, we cannot rule out the possibility that using e‐cigarettes makes cigarette smokers more dependent. Longitudinal data could shed light on the direction of causation.

## Conclusion

Among US high school students, recent increases in the prevalence of tobacco product use have not been accompanied by similar increases in the population burden of nicotine dependence. This appears to be at least partly attributable to a population‐level shift in the most common product of choice from cigarettes (on which users are most dependent) to e‐cigarettes (on which users are less dependent). Continued monitoring of trends in youth nicotine dependence alongside changes in tobacco product use is warranted.

## Declaration of interests

J.B. has received unrestricted research funding from Pfizer, who manufacture smoking cessation medications. All authors declare no financial links with tobacco companies or e‐cigarette manufacturers or their representatives.

## Author Contributions

**Jamie Brown:** Conceptualization; data curation; investigation; supervision. **Sarah Jackson:** Conceptualization; data curation; investigation. **Martin Jarvis:** Conceptualization; data curation.

## Supporting information

**Table S1** Indices of fit for the regression models: trends in dependence over time
**Table S2** Trends in dependence 2012–2019: results of the linear model and best fitting regression models
**Table S3** Indices of fit for the regression models: trends in product use over time
**Table S4** Indices of fit for the piecewise regression models: trends in dependence and product use over time
**Table S5** Trends in dependence 2012–2019: results of the piecewise regression models
**Table S6** Percentage (with 95% confidence interval) of high school students reporting wanting to first use tobacco products within 60 minutes of waking by type of product used in past 30 days. Figures in italics give the estimated percentage of all high school students using that product in past 30 days.
**Table S7** Comparison of slopes for measures of dependence and tobacco product use: tests of interactions between variable (dependence *vs.* product use) and year on prevalence.
