# Supplementary material for: Dependence on nicotine in US high school students in the context of changing patterns of tobacco product use
Source: Addiction. 2021 Jan 22;116(7):1859–70. doi: 10.1111/add.15403 (PMC8436751; doi:10.1111/add.15403)
Supplement: Supplementary file 1 — Table S1 Indices of fit for the regression models: trends in dependence over time Table S2 Trends in dependence 2012–2019: results of the linear model and best fitting regression models Table S3 Indices of fit for the regression models: trends in product use over time Table S4 Indices of fit for the piecewise regression models: trends in dependence and product use over time Table S5 Trends in dependence 2012–2019: results of the piecewise regression models Table S6 Percentage (with 95% confidence interval) of high school students reporting wanting to first use tobacco products within 60 minutes of waking by type of product used in past 30 days. Figures in italics give the estimated percentage of all high school students using that product in past 30 days. Table S7 Comparison of slopes for measures of dependence and tobacco product use: tests of interactions between variable (dependence vs. product use) and year on prevalence. [file ADD-116-1859-s001.docx]

| **Supplementary Table 1.** Indices of fit for the regression models: trends in dependence over time | | | | | | |
| --- | --- | --- | --- | --- | --- | --- |
|  | **Past 30-day craving for tobacco products** | | | **Wanting to first use tobacco products within 30 minutes of waking** | | |
| **Model** | **AIC** | **BIC** | **Adjusted R^2^** | **AIC** | **BIC** | **Adjusted R^2^** |
| Linear | 26.2 | 26.5 | 0.25 | 25.9 | 26.1 | -0.17 |
| Quadratic | 23.1 | 23.4 | 0.53 | 16.5 | 16.9 | 0.66 |
| Cubic | 21.8 | 22.2 | 0.61 | 15.7 | 16.1 | 0.70 |
| Logarithmic | 26.2 | 26.5 | 0.26 | 25.6 | 25.9 | -0.13 |
| Exponential | 27.5 | 27.8 | 0.21 | 25.6 | 25.9 | -0.17 |
| Power | 27.5 | 27.8 | 0.21 | 25.3 | 25.5 | -0.12 |
| AIC, Akaike Information Criterion; BIC, Bayesian Information Criterion. Shading indicates selected models. | | | | | | |

| **Supplementary Table 2.** Trends in dependence 2012-2019: results of the linear model and best fitting regression models | | | | | | | | |
| --- | --- | --- | --- | --- | --- | --- | --- | --- |
|  | **Past 30-day craving for tobacco products** | | | | **Wanting to first use tobacco products within 30 minutes of waking** | | | |
|  | **β** | **Lower 95% CI** | **Upper 95% CI** | ***p*** | **β** | **Lower 95% CI** | **Upper 95% CI** | ***p*** |
| **Linear model** |  |  |  |  |  |  |  |  |
| Intercept | 10.42 | 8.85 | 11.98 | <0.001 | 4.02 | 2.49 | 5.55 | 0.001 |
| Time | -0.28 | -0.66 | 0.09 | 0.115 | 0.01 | -0.36 | 0.38 | 0.951 |
| **Best fitting model** |  |  |  |  |  |  |  |  |
| Intercept | 10.88 | 9.00 | 12.75 | <0.001 | 4.86 | 3.58 | 6.14 | <0.001 |
| Time | -0.03 | -2.53 | 2.48 | 0.977 | -0.39 | -2.09 | 1.32 | 0.565 |
| Time^2^ | -0.31 | -1.18 | 0.56 | 0.374 | -0.12 | -0.71 | 0.48 | 0.615 |
| Time^3^ | 0.04 | -0.04 | 0.12 | 0.225 | 0.03 | -0.03 | 0.08 | 0.260 |

| **Supplementary Table 3.** Indices of fit for the regression models: trends in product use over time | | | |
| --- | --- | --- | --- |
|  | **Past 30-day use of any tobacco product** | | |
| **Model** | **AIC** | **BIC** | **Adjusted R^2^** |
| Linear | 46.8 | 47.1 | 0.10 |
| Quadratic | 44.8 | 45.1 | 0.35 |
| Cubic | 40.6 | 41.0 | 0.62 |
| Logarithmic | 47.9 | 48.1 | -0.02 |
| Exponential | 46.7 | 47.0 | 0.04 |
| Power | 47.5 | 47.8 | -0.06 |
| AIC, Akaike Information Criterion; BIC, Bayesian Information Criterion. Shading indicates selected model. | | | |

| **Supplementary Table 4.** Indices of fit for the piecewise regression models: trends in dependence and product use over time | | | | | | | |  | |  | |  | |
| --- | --- | --- | --- | --- | --- | --- | --- | --- | --- | --- | --- | --- | --- |
|  | **Past 30-day craving for tobacco products** | | | **Wanting to first use tobacco products within 30 minutes of waking** | | | **Past 30-day tobacco product use** | | | | | |  |
| **Model** | **AIC** | **BIC** | **Adjusted R^2^** | **AIC** | **BIC** | **Adjusted R^2^** | **AIC** | | **BIC** | | **Adjusted R^2^** | |  |
| Piecewise regression, breakpoint at 2017 | 16.7 | 17.1 | 0.80 | 15.1 | 15.5 | 0.73 | 38.5 | | 38.9 | | 0.71 | |  |
| AIC, Akaike Information Criterion; BIC, Bayesian Information Criterion. | | | | | | | |  | |  | |  | |

| **Supplementary Table 5.** Trends in dependence 2012-2019: results of the piecewise regression models | | | | | | | | | | |
| --- | --- | --- | --- | --- | --- | --- | --- | --- | --- | --- |
|  | **Past 30-day craving for tobacco products** | | | **Wanting to first use tobacco products within 30 minutes of waking** | | | **Past 30-day tobacco product use** | | | |
| **Model** | **β** | **Lower 95% CI** | **Upper 95% CI** | **β** | **Lower 95% CI** | **Upper 95% CI** | **β** | **Lower 95% CI** | **Upper 95% CI** |  |
| Slope 1: 2012-2017 | -0.66 | -1.11 | -0.20 | -0.39 | -0.80 | 0.02 | -0.61 | -1.96 | 0.74 |  |
| Slope 2: 2017-2019 | 1.01 | -0.01 | 2.03 | 1.25 | 0.33 | 2.17 | 5.32 | -2.66 | 13.31 |  |
|  | | | | | | | | | | |

| **Supplementary Table 6.** Percentage (with 95% confidence interval) of high school students reporting wanting to first use tobacco products within 60 minutes of waking by type of product used in past 30 days. Figures in italics give the estimated percentage of all high school students using that product in past 30 days. | | | | | | | | | | |
| --- | --- | --- | --- | --- | --- | --- | --- | --- | --- | --- |
|  | | **No product**  **used in p30 days** | **e-cigarettes**  **only** | **Smokeless, but no combustible** | | **Combustible, no cigarettes** | | **Cigarettes** | | **Total** |
|  | |  |  | **No e-cigs** | **+ e-cigs** | **No e-cigs** | **+ e-cigs** | **No e-cigs** | **+ e-cigs** |  |
| 2012 (*n*=12899) | | 0.3 (0.2-0.5) | 4.0 (0.5-25.4) | 17.7 (12.3-24.7) |  | 7.9 (5.2-11.7) | 25.1 (11.5-46.4) | 35.9 (32.2-39.7) | 54.6 (47.5-61.6) | 6.5 (5.6-7.5) |
| % population | | *76.9 (75-79)* | *0.2 (0.2-0.4)* | *1.8 (1.4-2.4)* | *0.0 (0.0-0.1)* | *7.0 (6.3-7.9)* | *0.3 (0.2-0.5)* | *11.5 (10.2-12.8)* | *2.2 (1.7-2.7)* |  |
| 2013 (*n*=10190) | | 0.5 (0.4-0.7) | 6.0 (2.1-15.6) | 27.4 (18.4-38.8) | 59.4 (21.4-88.7) | 7.5 (5.2-10.8) | 18.0 (7.9-35.8) | 39.9 (35.4-44.6) | 44.8 (36.5-53.5) | 6.6 (5.6-7.8) |
| % population | | *77.4 (76-79)* | *0.6 (0.5-0.8)* | *1.6 (1.1-2.2)* | *0.0 (0.0-0.1)* | *7.3 (6.5-8.2)* | *0.8 (0.6-1.1)* | *9.4 (8.2-10.7)* | *2.9 (2.4-3.5)* |  |
| 2014 (*n*=11399) | | 0.3 (0.2-0.5) | 1.4 (0.7-2.7) | 21.1 (14.6-29.4) | 20.0 (11.2-33.1) | 5.6 (4.0-7.9) | 10.3 (7.7-13.6) | 32.2 (26.8-38.0) | 40.2 (35.4-45.2) | 4.6 (4.0-5.4) |
| % population | | *75.5 (73-78)* | *4.4 (3.3-5.7)* | *1.5 (1.2-2.0)* | *0.6 (0.4-0.8)* | *5.7 (4.9-6.7)* | *3.3 (2.7-4.0)* | *4.0 (3.2-4.9)* | *5.0 (4.2-6.0)* |  |
| 2015 (*n*=9433) | | 0.3 (0.2-0.5) | 3.1 (1.7-5.8) | 35.9 (26.1-47.1) | 17.4 (7.2-36.6) | 4.6 (2.8-7.4) | 10.1 (6.7-14.9) | 27.8 (21.9-34.5) | 43.6 (37.8-49.6) | 4.9 (3.9-6.1) |
| % population | | *75.0 (73-77)* | *5.8 (4.8-7.0)* | *1.5 (1.1-2.3)* | *0.7 (0.5-0.9)* | *3.7 (3.2-4.4)* | *4.4 (3.7-5.1)* | *4.0 (3.2-5.1)* | *4.9 (4.0-5.9)* |  |
| 2016 (*n*=10,972) | | 0.2 (0.2-0.4) | 4.0 (2.2-7.3) | 24.5 (15.8-35.9) | 32.9 (17.3-53.4) | 9.8 (7.0-13.6) | 17.2 (12.0-24.0) | 39.9 (32.8-47.4) | 47.5 (40.4-54.8) | 4.9 (4.0-6.0) |
| % population | | *79.7 (78-82)* | *4.5 (3.7-5.4)* | *1.3 (1.0-1.7)* | *0.5 (0.4-0.8)* | *4.3 (3.7-5.0)* | *2.1 (1.8-2.6)* | *3.7 (2.9-4.7)* | *3.8 (3.2-4.7)* |  |
| 2017 (*n*=10,183) | | 0.2 (0.1-0.4) | 4.1 (2.2-7.5) | 26.2 (16.3-39.1) | 30.5 (13.2-55.9) | 10.1 (6.2-16.1) | 19.5 (11.4-31.2) | 32.5 (25.6-40.1) | 38.2 (31.9-44.9) | 4.1 (3.2-5.3) |
| % population | | *80.3 (78-83)* | *5.3 (4.2-6.6)* | *0.9 (0.7-1.3)* | *0.4 (0.2-0.6)* | *3.8 (3.3-4.5)* | *2.0 (1.5-2.8)* | *3.5 (2.9-4.3)* | *3.8 (3.2-4.6)* |  |
| 2018 (*n*=10,991) | | 0.3 (0.2-0.5) | 10.8 (8.2-14.2) | 24.7 (16.9-34.7) | 35.6 (24.7-48.2) | 8.4 (5.3-13.0) | 22.5 (18.5-27.1) | 34.2 (26.8-42.3) | 42.7 (37.9-47.6) | 6.1 (5.3-7.0) |
| % population | | *72.8 (71-75)* | *11.1 (9.6-13.1)* | *1.0 (0.7-1.3)* | *0.9 (0.7-1.3)* | *3.4 (2.8-4.1)* | *3.0 (2.5-3.5)* | *2.4 (2.0-2.9)* | *5.4 (4.6-6.4)* |  |
| 2019 (*n*=10,097) | | 1.0 (0.2-5) | 13.8 (11.6-16.3) | 35.4 (19.9-54.6) | 29.2 (20.8-39.4) | 14.1 (9.1-21.1) | 28.6 (23.2-34.6) | 25.0 (14.8-39.0) | 48.7 (43.1-54.3) | 7.8 (5.9-10.1) |
| % population | | *68.7 (67-71)* | *17.2 (15.6-19)* | *0.8 (0.5-1.2)* | *1.0 (0.7-1.4)* | *2.4 (1.8-3.2)* | *4.2 (3.7-4.8)* | *0.8 (0.6-1.2)* | *4.9 (3.8-6.3)* |  |

| **Supplementary Table 7.** Comparison of slopes for measures of dependence and tobacco product use: tests of interactions between variable (dependence vs. product use) and year on prevalence | | | | | | | | | |
| --- | --- | --- | --- | --- | --- | --- | --- | --- | --- |
|  | **Past 30-day craving for tobacco products**  **vs. past 30-day tobacco product use** | | | | **Wanting to first use tobacco products within 30 minutes of waking**  **vs. past 30-day tobacco product use** | | | |  |
|  | **β** | **Lower 95% CI** | **Upper 95% CI** | ***p*** | **β** | **Lower 95% CI** | **Upper 95% CI** | ***p*** |  |
| Slope 1: 2012-2017 | -0.04 | -1.14 | 1.06 | 0.931 | 0.29 | -0.82 | 1.41 | 0.559 |  |
| Slope 2: 2017-2019 | -4.65 | -9.96 | 0.66 | 0.064 | -4.55 | -9.04 | -0.06 | 0.049 |  |
